# Recent Advances in Mechanically Loaded Human Mesenchymal Stem Cells for Bone Tissue Engineering

**DOI:** 10.3390/ijms21165816

**Published:** 2020-08-13

**Authors:** Kar Wey Yong, Jane Ru Choi, Jean Yu Choi, Alistair C. Cowie

**Affiliations:** 1 Department of Surgery, Faculty of Medicine & Dentistry, University of Alberta, Edmonton, AB T6G 2R3, Canada; 2 Department of Mechanical Engineering, University of British Columbia, 2054-6250 Applied Science Lane, Vancouver, BC V6T 1Z4, Canada; 3 Centre for Blood Research, Life Sciences Centre, University of British Columbia, 2350 Health Sciences Mall, Vancouver, BC V6T 1Z3, Canada; 4 Ninewells Hospital & Medical School, Dundee, Scotland DD1 5EH, UK; j.y.choi@dundee.ac.uk (J.Y.C.); Acowie001@dundee.ac.uk (A.C.C.)

**Keywords:** bone repair, mechanical loading, human mesenchymal stem cells, bone tissue engineering, mechanotransduction

## Abstract

Large bone defects are a major health concern worldwide. The conventional bone repair techniques (e.g., bone-grafting and Masquelet techniques) have numerous drawbacks, which negatively impact their therapeutic outcomes. Therefore, there is a demand to develop an alternative bone repair approach that can address the existing drawbacks. Bone tissue engineering involving the utilization of human mesenchymal stem cells (hMSCs) has recently emerged as a key strategy for the regeneration of damaged bone tissues. However, the use of tissue-engineered bone graft for the clinical treatment of bone defects remains challenging. While the role of mechanical loading in creating a bone graft has been well explored, the effects of mechanical loading factors (e.g., loading types and regime) on clinical outcomes are poorly understood. This review summarizes the effects of mechanical loading on hMSCs for bone tissue engineering applications. First, we discuss the key assays for assessing the quality of tissue-engineered bone grafts, including specific staining, as well as gene and protein expression of osteogenic markers. Recent studies of the impact of mechanical loading on hMSCs, including compression, perfusion, vibration and stretching, along with the potential mechanotransduction signalling pathways, are subsequently reviewed. Lastly, we discuss the challenges and prospects of bone tissue engineering applications.

## 1. Introduction

Bone is a specialized hard tissue composed of bone cells (e.g., osteoblasts, osteocytes and osteoclasts) embedded in a mineralized organic matrix, consisting mainly of calcium phosphate, collagen and water [1]. It generally protects and supports various organs in the body, enabling mineral storage and body movement, and can be self-regenerated in response to injury or defect due to its intrinsic regenerative capability [2]. Small bone defects usually heal with the formation of new bone, with no to minimal scar tissue. However, large bone defects (fracture gap more than 2.5 cm) such as osteoporosis and osteonecrosis caused by trauma, tumour or infection require a larger-scale bone regeneration, which is beyond the self-healing capacity of a normal bone, thereby necessitating external bone repair approaches [3,4,5]. 

To date, there are no reliable long-term healing approaches for the repair of large bone defects. The conventional clinical approaches used to augment bone regeneration include distraction osteogenesis-and-bone transport, bone grafting and Masquelet techniques [6,7]. Among these, bone grafting is the current gold-standard approach for the repair of non-healing or large bone defects. Autograft bone is more attractive owing to its intrinsic integration and compatibility, yet its use has numerous restrictions, including its limited availability and donor site morbidity [8]. Allogeneic bone is less preferable due to the risk of donor-to-host disease transmission and graft rejection [9]. On the other hand, distraction osteogenesis-and-bone transport is a technique that induces spontaneous bone regeneration by means of tensile strain. However, the risks of complications such as infection are high due to the need for an external fixator and a series of subsequent surgeries to achieve optimal bone healing [10]. To accelerate bone regeneration, the Masquelet technique incorporates induced membranes into cancellous autograft bones to revascularize the bone graft and prevent bone resorption. Nevertheless, the risk of complications associated with the use of an external fixator and multiple surgeries are similarly high [7]. The limitations of conventional techniques therefore present a strong demand for alternative bone regeneration approaches that address these drawbacks.

Bone tissue engineering using tissue-engineered bone grafts derived from human mesenchymal stem cells (hMSCs) offers a promising alternative solution for the treatment of large bone defects. These cells can be isolated from various tissues in the human body, are easily propagated in vitro, and can be induced to differentiate into mineral-producing osteocytes, leading to new bone formation [11,12]. Several studies showed that hMSC-laden porous scaffolds are more effective than the cell-free scaffolds in mediating host cell integration, with new bone being formed within two months of implantation in animal bone defect models (e.g., rat and mouse) [13,14]. Moreover, recent clinical studies also demonstrated promising osteogenic outcomes in patients with bone defects receiving tissue-engineered bone grafts derived from hMSCs [15,16,17]. For instance, through X-rays, one study revealed that most patients with bone defects had good bone healing within four months of receiving hMSC-laden scaffolds [18]. However, some patients suffered from lack of bone healing possibly due to osteolysis and resorption of hMSC-derived bone grafts generated using static culture [18,19]. As the static culture only allows a slow rate of diffusion of oxygen and nutrients to the central core of scaffolds, many hMSCs situated there were reported to undergo apoptosis or necrosis due to insufficient nutrient and oxygen supplies [20,21]. Lack of viable and functional cells consequently led to inadequate bone matrix formation and poor mechanical strength of the bone grafts. To address these concerns, dynamic culture using a bioreactor that provides mechanical loading was introduced in the development of large tissue-engineered bone grafts with clinically relevant size (length of the grafts should be more than 2.5 cm, based on the size of large bone defects [22]). Several studies have used perfusion bioreactors to create tissue-engineered bone grafts derived from animal MSCs (e.g., sheep and goat) and implanted the grafts into animal bone defect models (e.g., sheep and goat) [21,23,24]. The number of cells, the uniform cell distribution, and the amount of new bone matrix in the scaffolds were greatly enhanced, as perfusion provides sufficient nutrient and oxygen to the cells by supporting the interstitial flow of cell culture medium throughout the porous scaffolds. Six months after the implantation in the large-animal bone defect models, it was found that the bioreactor-generated bone grafts induced more new bone formation compared static-culture-generated bone grafts or cell-free scaffolds [21,23,24]. This evidence suggests that mechanical loading is a key component in bone tissue engineering, given that native bone is constantly subjected to mechanical loading in daily activities [25]. 

Recent studies have shown that mechanical loading (e.g., compression, perfusion, vibration and stretching) is effective in enhancing the osteogenesis and bone mineralization of hMSCs compared to static-cultured hMSCs [8,26,27,28]. For instance, a dynamic biaxial compression bioreactor was developed and applied on hMSC-laden three-dimensional (3D) scaffolds, and results demonstrated its capability of increasing the expression of osteogenic markers (e.g., alkaline phosphatase (ALP), osteonectin (ON) and osteocalcin (OCN)) in hMSCs [26]. Another study applied perfusion to hMSC-laden scaffolds using an oscillatory perfusion bioreactor, demonstrating its potential in enhancing OCN expression and calcium deposition in hMSCs [8]. However, the understanding of the effects of specific types of mechanical loading, loading regime, and mechanotransduction signalling on osteogenesis remains poor. Therefore, exploring these properties will help in establishing a functional tissue-engineered bone graft for clinical applications.

There are several reviews on the effects of mechanical loading on matrix production and differentiation (e.g., tenogenesis, myogenesis, chondrogenesis and osteogenesis) of MSCs derived from animals and humans [29,30,31]. However, the effects of various types of mechanical loading on the osteogenic differentiation of hMSCs for bone tissue engineering have not been comprehensively reviewed. The escalating demand for tissue-engineered bone grafts presents a strong need for a comprehensive review on this topic covering studies published over the past decade. We first highlight the key assays for assessing the quality of tissue-engineered bone grafts, including special staining, as well as gene and protein expression of osteogenic markers. The studies published over the last 10 years on the mechanical loading effects on hMSCs, including compression, perfusion, vibration and stretching, along with the potential mechanotransduction signalling for the osteogenesis of hMSCs are subsequently reviewed. Finally, the challenges associated with the future translation of hMSC-derived bone grafts to large bone defect therapy are discussed.

## 2. Key Assays for Assessing the Quality of Tissue-Engineered Bone Grafts

In general, the quality of tissue-engineered bone grafts can be assessed by several key tests, including osteogenic protein or gene expression assays and specific staining. Firstly, an ideal tissue-engineered bone graft for bone defect repair should present the osteogenic phenotypes. The osteogenic phenotypes of MSCs upon osteogenic induction can be evaluated by analysing the gene and protein expression of osteogenic markers, including alkaline phosphatase (ALP), runt-related transcription factor 2 (RUNX2), osteocalcin (OCN), osteopontin (OPN), osterix (OSX), osteonectin (ON) and bone sialoprotein 2 (BSP). The common tests used are quantitative real-time polymerase chain reaction assay, Western blotting, as well as immunofluorescent and immunohistochemical staining. In fact, the osteogenic ability of MSCs is mainly regulated by two transcription factors, RUNX2 and OSX [32]. RUNX2 is a protein that binds to its response elements (specific DNA sequences within its target genes) to regulate the transcription of its target genes [33]. RUNX2 is known as a master regulator of osteogenic differentiation. It binds to osteoblast-specific *cis*-acting element 2 (OSE2), which is present in the promoter region of several osteogenic genes including OPN, ON, OCN and BSP, leading to an increase in the transcription rate or expression of these genes [33,34]. Similarly, OSX is a zinc-finger-containing protein that binds to the promoter region of the osteogenic genes to upregulate the transcription of these genes [35,36]. Upon the osteogenic induction of MSCs, ALP, an early osteogenic marker, plays a critical role in phosphate and calcium deposition for bone mineralization [37]. Apart from protein and gene expression assay, the ALP activity of MSCs can also be assessed through a colorimetric ALP assay [26]. OPN contributes to bone mineralization and remodelling [38], whereas ON can strongly bind to calcium phosphate and collagen to enhance the mineralization of bone matrix [39]. Bone matrix synthesis and mineralization are controlled by OCN, which acts as a late osteogenic marker [37]. BSP-2 is another late osteogenic marker that enhances ALP activity and OCN synthesis [40]. Enhanced expression of these osteogenic markers has been observed upon osteogenic induction of MSCs in the presence of mechanical or biochemical cues [28,41]. In short, the early osteogenesis of MSCs means the differentiation of MSCs into the early stage of osteoblast, which is indicated by a higher expression of early osteogenic markers (RUNX2 and ALP) compared to undifferentiated MSCs. Late osteogenesis of MSCs means the differentiation of MSCs into the late stage of osteoblast, which is indicated by a higher expression of late osteogenic markers (OCN and BSP) and the deposition of calcium compared to undifferentiated MSCs [42,43]. 

Secondly, an ideal tissue-engineered bone graft should high amounts of bone minerals, especially calcium. Specific stains such as Alizarin Red and Von Kossa are mainly used to detect calcium and phosphate, respectively, within a tissue-engineered bone graft derived from MSCs [28,44]. In addition to special stains, scanning electron microscopy and Raman spectroscopy can also be performed to evaluate bone mineralization [26,28]. Moreover, the quantification of calcium deposits can be done through a colorimetric assay [45]. Besides calcium detection, the mechanical properties (e.g., Young’s modulus and bone volume fraction) of a tissue-engineered bone graft should be determined [8,45]. Apart from in vitro assays, a tissue-engineered bone graft derived from MSCs can be subcutaneously implanted into non-obese diabetic-severe combined immunodeficiency interleukin-2 receptor gamma chain gene mice to evaluate its capability of mediating in vivo bone formation and host tissue integration [8]. Micro-computed tomography and X-ray are good methods for the real-time monitoring of bone healing in both human and animal bone defect models receiving tissue-engineered bone grafts [18,46]. 

## 3. Effects of Mechanical Loading on Osteogenesis of hMSCs

**Figure 1:**
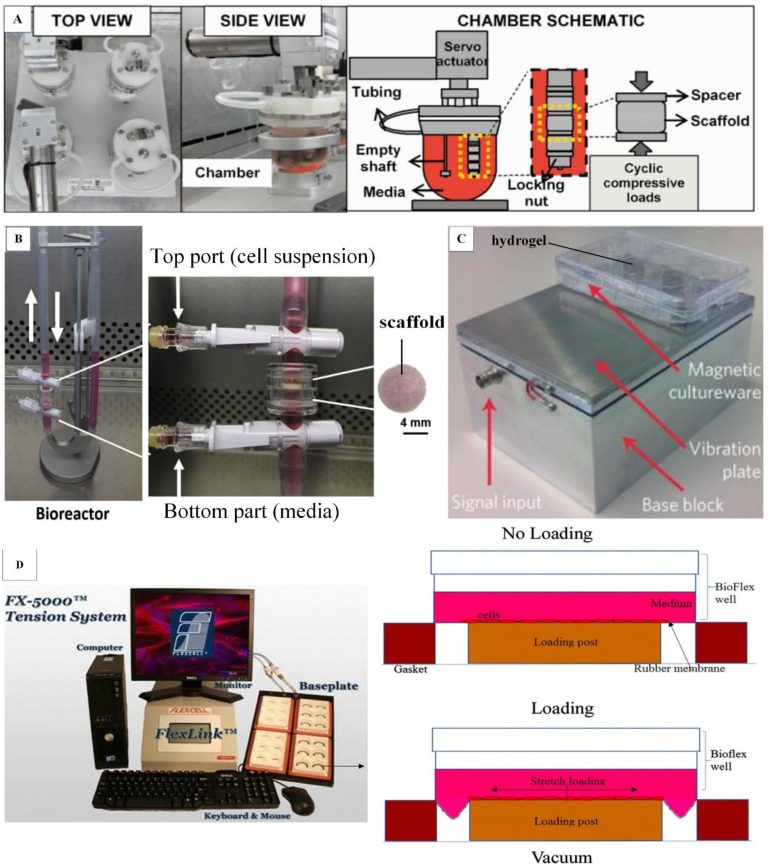
Bioreactors for bone tissue engineering. (**A**) Compression bioreactor. The locking nut tightens the scaffolds to the shaft of the chamber lid and support the scaffolds from the bottom. During loading, the locking nut and a micromanipulator on the top of the chamber compress the scaffolds simultaneously. Adapted with permission from [26] © John Wiley & Sons (2017). (**B**) Oscillatory perfusion bioreactor. Perfusion rate is controlled by a syringe pump connected to the perfusion bioreactor. Adapted with permission from [8] © Elsevier (2017). (**C**) Vibrational bioreactor. The vibration plate has a layer of magnetic stainless steel that allows a magnetic well plate to be adhered firmly on the vibration plate. This provides extra rigidity to ensure the consistent transfer of vibration amplitude across each well. Adapted with permission from [28] © Springer Nature (2017). (**D**) Stretching device. During loading, the cells cultured on the rubber membrane are stretched when the membrane deforms downwards under the negative pressure. Adapted with permission from [62] © Creative Commons Attribution License (2020).

hMSCs are commonly seeded in a 3D porous scaffold (e.g., ceramics, demineralized bone matrix and synthetic polymers) for the development of tissue-engineered bone grafts. hMSCs derived from different sources, such as bone marrow, fat, umbilical cord blood, periosteum, mandibular retromolar bone and periodontal ligament, have been used in bone tissue engineering [27,44,47,48,49]. Notably, bone-marrow-derived hMSCs are extensively used in bone tissue engineering due to their relatively high osteogenic potential compared to other sources of hMSCs [50]. A scaffold with an interconnected porous structure (pore sizes range from 100 to 800 μm) allows efficient nutrient diffusion, cell ingrowth and migration [51]. Ceramic scaffolds (e.g., hydroxyapatite and β-tricalcium phosphate (β–TCP)) and demineralized bone matrix have been widely used in bone tissue engineering due to their osteoinductive, osteoconductive and osseointegrative properties [52,53]. However, ceramics present uncontrollable brittleness and difficulty of shaping for implantation, whereas demineralized bone matrix displays low initial mechanical strength, restricting its use for repairing large bone defects [54,55]. Alternatively, synthetic polymers such as poly-lactic-co-glycolic acid (PLGA) and poly-l-lactic acid (PLA) have been introduced because their mechanical properties and porous architectures can be easily tuned to recapitulate those of native bone by simply manipulating the composition and synthesis method of the polymers [56]. Moreover, their degradation rate can be tuned to meet the pace of new bone tissue formation and host cell integration [57]. However, they show moderate osteoconductivity, and the degradation of some synthetic polymers may create an acidic microenvironment that is harmful to tissue [57,58]. Therefore, some studies have developed hybrid scaffolds for bone tissue engineering in order to maximize the benefits of both ceramics and synthetic polymers [8,26]. Traditionally, hMSC-laden scaffolds were subjected to biochemical cues (e.g., dexamethasone and β-glycerophosphate) to develop tissue-engineered bone grafts. Although the grafts have expressed bone-specific proteins, they lacked sufficient mechanical properties to withstand physiological loading [59]. Therefore, various types of mechanical loading, such as compression (Figure 1A), perfusion (Figure 1B), vibration (Figure 1C) and stretching (Figure 1D), can be applied to hMSC-laden scaffolds using bioreactors to improve the mechanical properties of tissue-engineered bone grafts [28,60,61,62]. Selected recent in vitro studies that explored the effects of mechanical loading on the osteogenesis of hMSCs in two-dimensional (2D) planar culture and 3D scaffold are summarized in Table 1 and Table 2, respectively. The findings from 2D studies may provide a good platform for guiding 3D studies to elucidate mechanotransduction pathways in the osteogenic differentiation of hMSCs. Most studies applied mechanical loading to undifferentiated hMSCs, and only a few studies applied mechanical loading to osteogenically differentiated hMSCs (precultured in osteogenic induction medium) (Table 1 and Table 2).

### 3.1. Compression

Human bone is physiologically subjected to compressive strain and fluid shear stress for bone remodelling [25]. Compressive strain is imposed on osteocytes through the compression and relaxation of bone extracellular matrix, which is caused by cyclic physiological loading and unloading. This is resulted from muscular contraction due to daily activities [25,78]. It has been reported that the physiological compressive strain experienced by bone cells is between 0.2% and 0.4% [79]. Strain below this range caused by prolonged muscle disuse may lead to bone resorption, whereas strain above this range due to exercise may strengthen the bone. For example, people who are having spinal cord injury or space travel tend to develop bone loss, whereas denser bones can be seen in the arms of professional tennis players [80]. 

**Figure 2:**
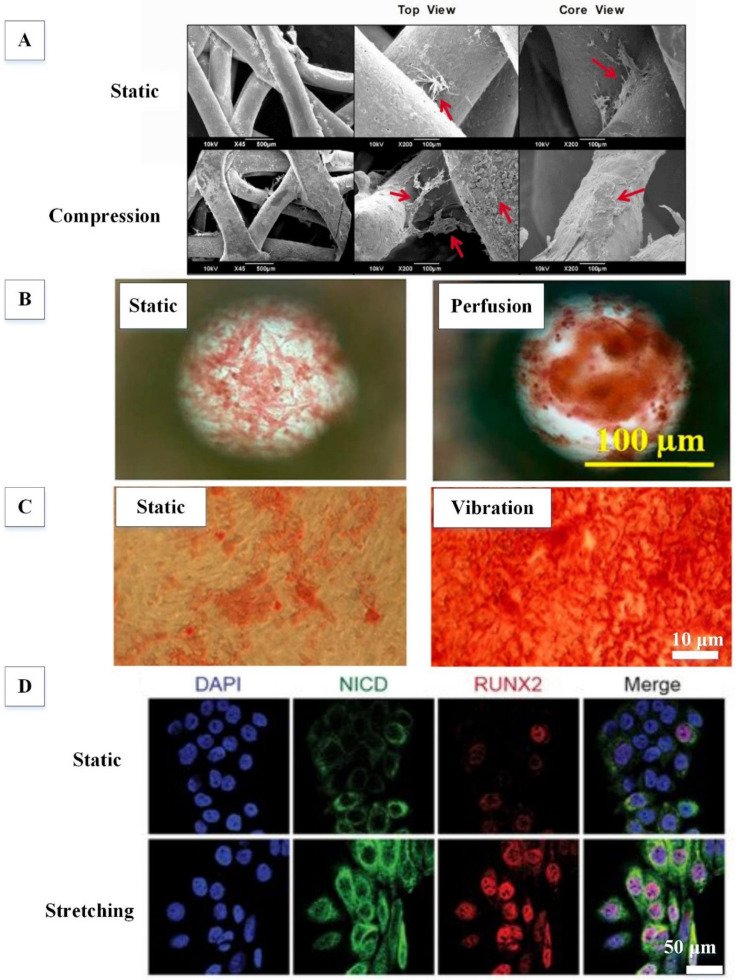
Effects of mechanical loading on the osteogenesis of human mesenchymal stem cells (hMSCs). (**A**) Dynamic compression improved bone mineralization. Red arrows show mineral deposits. Adapted with permission from [26] © John Wiley & Sons (2017). (**B**) Perfusion enhanced calcium deposition, as indicated by an increase in percentage of Alizarin-Red-stained area. Adapted with permission from [47] © ACS Publications (2018). (**C**) Vibration increased calcium deposition, as indicated by an increase in the percentage of Alizarin-Red-stained area. Adapted with permission from [69] © Creative Commons Attribution License (2013). (**D**) Dynamic stretching promoted the expression of RUNX2 by increasing the expression of Notch intracellular domain (NICD), which transcriptionally activates the Notch signalling pathway. Adapted with permission from [65] © Creative Commons Attribution License (2017).

Dynamic compression has been applied to hMSC-laden scaffolds at strains ranging from 0.22% to 20% using compression bioreactors to understand the effects of compression on the osteogenic potential of hMSCs [26,60,71]. Interestingly, it was found that a short-term uniaxial compression (0.4%, 0.1 Hz, 2 h/day for 1 day) on hMSC-laden monetite calcium phosphate scaffold was sufficient to enhance the expression of the osteogenic transcription factor RUNX2 [60]. This study suggests that the extension of loading duration might lead to further activation of many genes involved in the osteogenic differentiation of hMSCs. Toward this end, another study applied long-term dynamic biaxial compression (0.22–1.1%, 1 Hz, 4 h/day for 4 weeks) on hMSCs seeded into a hybrid polycaprolactone/β-TCP scaffold [26]. Among the selected strains, it was found that 0.22% was better than 0.88% and 1.1% in enhancing ALP activity of hMSCs. Additionally, mineral deposition (Figure 2A) as well as ON and OCN expression were found to be higher in hMSCs subjected to 0.22% strain compared static controls. Moreover, two recent studies have also shown that the osteogenic potential of hMSCs is reduced by uniaxial compressive loading in a strain-dependent manner [71,72]. Static control has a higher expression of osteogenic markers (RUNX2, ON and OCN) and calcium deposition than hMSCs treated with a dynamic compressive strain in the range of 5% to 20%. In fact, such compressive strains are more efficient in enhancing the chondrogenic potential of hMSCs than their osteogenic potential [81]. Based on the aforementioned literature, we suggest that the loading regime of a biaxial or uniaxial compression bioreactor should be set as follows: a compressive loading amplitude of 0.2–0.4% strain, a loading frequency of 1 Hz, and a loading duration of 4 weeks (4 h/day). Further studies are required to evaluate the mechanical properties of hMSC-laden scaffolds subjected to dynamic compression and their potential use in animal pre-clinical studies prior to clinical trials. 

### 3.2. Perfusion

Fluid shear stress is predominantly imposed on osteocytes and MSCs [82,83]. hMSCs in 2D planar culture have been subjected to fluid shear stress (0.12–12 dyn/cm^2^) through perfusion to assess their osteogenic potential [47,63,64]. It was found that a short-term continuous, unidirectional perfusion (0.12–0.15 dyn/cm^2^, 4 days; 12 dyn/cm^2^, 3 days) was sufficient to induce the early osteogenesis of hMSCs, as indicated by an increased expression of ALP, RUNX2 and OSX [63,64]. Moreover, a long-term continuous, unidirectional perfusion (10 dyn/cm^2^, 3 weeks) was able to enhance the expression of RUNX2 and induce late osteogenesis, as evidenced by an increased expression of OCN and calcium deposition (Figure 2B) [47]. 

To generate fluid shear stress for the osteogenesis of hMSCs cultivated in 3D porous scaffold, many efforts have been devoted to the development of perfusion bioreactors. Besides generating fluid shear stress, perfusion can also improve oxygen and nutrient transport to cells cultured in large 3D scaffolds via convection and enhances waste removal, resulting in excellent cell distribution, growth and osteogenic differentiation within the scaffold [84,85]. Moreover, perfusion bioreactors can also provide various fluid flow profiles, such as unidirectional, pulsatile or oscillatory flow [8,45,61]. The fluid shear stress given by such fluid flow has been shown to enhance the osteogenic differentiation of hMSCs. For instance, one study applied a single, unidirectional perfusion (0.0679 dyn/cm^2^, 2 h) on early and late osteogenic differentiated hMSCs cultured in a porous gelatine-coated polyurethane scaffold (334 μm) [73]. This perfusion regime was able to further increase RUNX2 expression in early osteogenic differentiated hMSCs (precultured in osteogenic induction medium for 1 week) and enhance the expression of OCN in late osteogenic differentiated hMSCs (precultured in osteogenic induction medium for 15 days). Furthermore, another study applied a long-term continuous, unidirectional perfusion (0.161 dyn/cm^2^, 3 weeks) on hMSCs seeded in decellularized porcine bone scaffold (250–400 μm) [44]. It was found that calcium deposition and expression of RUNX2, OPN and OCN were higher in perfusion-treated hMSCs than in the static control.

Given that oscillatory or pulsatile flow is physiologically generated through daily activities and the circulatory system, some studies have applied oscillatory or pulsatile flow to hMSCs for the development of tissue-engineered bone grafts [8,45,74,75]. For example, one study applied a long-term, continuous, oscillatory perfusion (0.0123 dyn/cm^2^, 3 weeks) to hMSCs cultivated in a scaffold (200–800 μm) made of cancellous bone powder [74]. This loading regime was found to enhance early osteogenesis, as indicated by a higher expression of RUNX2 and ALP compared to the static control. Another study increased the oscillating shear stress (0.2 dyn/cm^2^, 2 weeks) on hMSCs seeded in a hybrid hyaluronic acid/PLGA scaffold (300 μm), resulting in an enhanced bone volume fraction (ratio of bone volume to total tissue volume) and an enhanced expression of OCN, which indicates late osteogenesis [8]. Additionally, blood vessels were formed in both the cell-laden and cell-free scaffolds, indicating that host tissues were integrated into the scaffolds to induce angiogenesis. Moreover, this perfusion-treated bone construct was implanted into non-obese diabetic-severe combined immunodeficiency interferon-2 receptor gamma chain gene mice, showing excellent in vivo bone formation capability as indicated by positive immunohistochemical staining for BSP. Apart from oscillatory flow, one study sequentially applied continuous, steady perfusion (0.045 dyn/cm^2^, 2 weeks) and intermittent pulsatile perfusion (0.045 and 0.134 dyn/cm^2^ at 0.5 Hz, 4 h/day for 3 weeks) on osteogenic differentiated hMSC-laden porous silk fibroin scaffolds (400–600 μm) (precultured in osteogenic induction medium for 3 days) [45]. This sequence of physiologically relevant fluid flow profile increased the expression of OPN and BSP in hMSCs, which were higher than in hMSCs subjected to steady perfusion only (0.045 dyn/cm^2^, 5 weeks). Following the perfusion, the Young modulus of these bone grafts was improved by 1.8-fold, from 150 to 270 kPa. The bone volume fraction of these bone grafts was also enhanced. Overall, perfusion was found to improve the osteogenesis of hMSCs at shear stresses ranging from 0.0182 to 4.2 dyn/cm^2^. However, similar osteogenic potential was observed in static-cultured hMSCs and hMSCs exposed to a low shear stress (e.g., 0.0097 dyn/cm^2^) [76]. These findings indicate that high fluid flow shear stress is beneficial for the osteogenesis of hMSCs.

Besides the perfusion regime, the osteogenic potential of perfusion-treated hMSCs is also dependent on the scaffold pore size. For instance, following perfusion with a flow rate of 1.5 mL/min, hMSCs cultivated in a scaffold with small pores (750 μm) expressed more ALP and OPN than those cultivated in a scaffold with large pores (1400 μm) [76]. Moreover, cell source may also affect the osteogenic potential of perfusion-treated hMSCs. For example, following perfusion with a flow rate of 1 mL/min, it was found that bone-marrow-derived hMSCs presented higher levels of osteogenic markers (e.g., RUNX2, OPN, ALP and calcium deposition) than adipose-derived hMSCs [44]. Based on the aforementioned literature, we suggest that the loading regime of a perfusion bioreactor should be set as follows: oscillatory or pulsatile flow, fluid flow shear stress >0.02 dyn/cm^2^ and loading duration >2 weeks. This should be performed on hMSCs cultivated in a 3D porous scaffold (100–800 μm) in conjunction with osteoinductive supplements to achieve optimal effects of perfusion on osteogenesis, producing a fully functional tissue-engineered bone graft. 

### 3.3. Vibration

In the past decade, human studies have suggested that low-magnitude, high-frequency vibration therapy delivers mechanical signals to osteoporosis patients to improve musculoskeletal strength by increasing bone formation [86,87]. Therefore, vibration has recently been introduced to induce osteogenesis in hMSCs in 2D planar culture and 3D scaffold. For instance, a short-term vibration therapy (0.3–0.6 × *g*, 50 Hz, 30 min/day for 5 days) was able to increase the expression of RUNX2, OSX, ALP and OCN in hMSCs cultured on a 2D extracellular matrix (ECM)-coated polystyrene substrate via a vibration sensor [49]. Besides upregulating the expression of osteogenic markers in hMSCs, a long-term vibration therapy (0.59 × *g*, 30 Hz, 45 min/day for 6 weeks) also increased calcium deposition (Figure 2C) [69]. In addition to vibratory magnitude or amplitude, one study optimized the vibratory frequency needed to augment the osteogenic potential of hMSCs [70]. This study demonstrated that hMSCs subjected to vibration at 800 Hz expressed more ALP, RUNX2 and OPN and secreted more calcium than those subjected to vibration at both 30 Hz and 400 Hz. For 3D study, a nanovibrational bioreactor was developed to apply vibration to hMSCs cultivated in a 3D collagen gel through a reverse piezo effect [28]. It was found that this vibration (30 nm amplitude, 1000 Hz, 7 days) enhanced the expressions of OSX, ALP, OPN and OCN in hMSCs without any osteoinductive supplements. Taken together, vibration could be a promising approach for the development of tissue-engineered bone grafts derived from hMSCs. Further studies are required to evaluate the effect of vibration on the osteogenic potential of hMSCs cultured in other types of scaffolds (e.g., ceramics, synthetic polymers and demineralized bone matrix). 

### 3.4. Stretching

Tensile strain is clinically used in distraction osteogenesis-and-bone transport [25]. In this bone repair technique, two bone segments are slowly stretched and moved apart from each other to create a fracture between the bone segments, allowing spontaneous bone regeneration. Therefore, it has been suggested that stretching may play a role in the osteogenesis of hMSCs. In one study, a short-term continuous dynamic biaxial stretching (4%, 0.5 Hz, 8 h) was applied to osteogenic differentiated hMSCs (precultured in osteogenic induction medium for 2 weeks) cultured on a 2D collagen-coated silicone membrane [48]. This loading regime was able to further enhance the expression of ALP and OCN, as well as calcium deposition in osteogenic differentiated hMSCs. Moreover, undifferentiated hMSCs subjected to long-term continuous dynamic biaxial stretching (10%, 0.5 Hz, 2–3 weeks) were found to increase the secretion of calcium and highly express RUNX2 (Figure 2D), ALP, ON, OPN and OCN [27,65]. Apart from continuous stretching, some studies have applied intermittent stretching to hMSCs to promote osteogenesis. For instance, a short-term intermittent dynamic stretching (3%, 0.2 Hz, 4 h/day for 4 days) was able to induce early osteogenesis of hMSCs cultivated on fibronectin-coated polydimethylsiloxane membrane, as indicated by an enhanced expression of RUNX2 [66]. When tensile strain and frequency was increased to 10% and 0.5 Hz, respectively, late osteogenesis of hMSCs was observed following the intermittent biaxial stretching (6 h/day for 7 days) [67]. Most of these 2D studies were performed using commercially available cyclic stretching devices such as Flexcell, which provides tunable tensile strain (1–33%) and a well-characterized strain profile [27,48,65,67]. Interestingly, one recent study applied tensile strain to hMSCs cultured on a TiO_2_ nanotubes substrate—a material which is widely used clinically as load-bearing metal implant. It was found that dynamic uniaxial stretching (0.9%, 5 Hz, 0.5 h/day for 7 days) promoted the expression of both early and late osteogenic markers, such as RUNX2, ALP, OPN, OCN and BSP [68]. These findings could provide some novel mechanotransduction pathways in the stretch-induced osteogenic differentiation of hMSCs. In the future, more studies are required to perform stretching on hMSC-laden 3D scaffold in order to validate the pathways.

## 4. Mechanotransduction Signalling for the Osteogenesis of hMSCs

**Figure 3:**
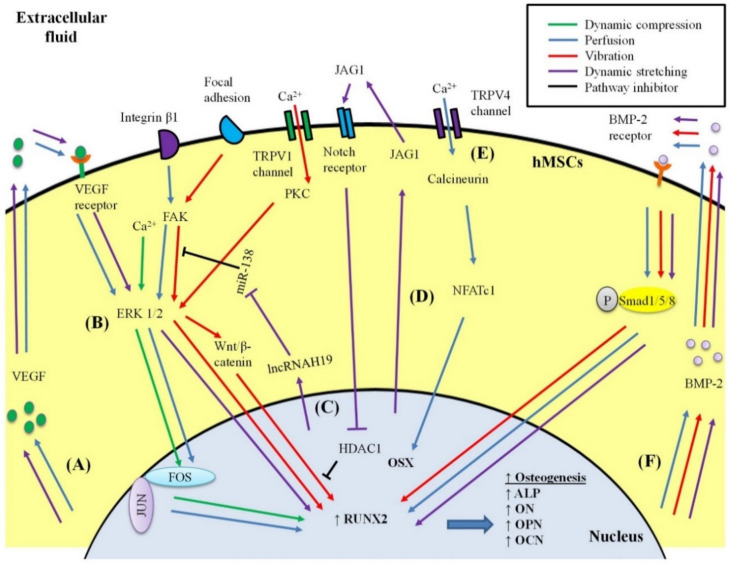
Mechanotransduction signalling for osteogenesis of human mesenchymal stem cells (hMSCs). (**A**) VEGF signalling pathway: VEGF is produced and released into extracellular fluid to bind to its receptor and activate the ERK 1/2-RUNX2 signalling pathway. (**B**) ERK 1/2-RUNX2 pathway: a common pathway shared by many types of mechanical loading to induce osteogenesis. (**C**) Dynamic stretching increases the production of long non-coding RNA H19 (lncRNAH19) to inhibit the function of miR-138 in blocking the FAK-ERK 1/2-RUNX2 signalling pathway. (**D**) Notch signalling pathway: dynamic stretching enhances the production and release of JAG1 into extracellular fluid, which binds to the Notch receptor to inhibit the role of HDAC1 in blocking the Wnt/β-catenin pathway involved in osteogenesis. (**E**) Through TRPV4 channel, perfusion activates calcineurin, which increases the nuclear translocation of NFATc1, which forms a complex with OSX to induce osteogenesis. (**F**) BMP-2 signalling pathway: BMP-2 is produced and released into extracellular fluid to bind to its receptor, which then enhances the nuclear translocation of phosphorylated Smad 1/5/8 for triggering osteogenesis.

Different types of mechanical loading may induce osteogenesis in hMSCs via similar or different metabolic routes. The proposed mechanisms of each mechanical loading in inducing osteogenesis of hMSCs are summarized in Figure 3. In brief, dynamic compression may activate calcium signalling that subsequently upregulates the phosphorylation of extracellular signal-regulated kinase (ERK) 1/2, thereby increasing the expression of FOSB in hMSCs [60]. FOSB protein and c-Jun protein then form a complex called activator protein-1 (AP-1) that readily binds to DNA, leading to an increase in the transcription rates of RUNX2 and other osteogenic genes.

Perfusion provides fluid shear stress, which may induce osteogenesis via several metabolic routes. Perfusion activates the mechanoreceptor integrin β1-mediated phosphorylation of focal adhesion kinase (FAK), resulting in the phosphorylation of ERK 1/2, which leads to the activation of RUNX2 and the transcription of other osteogenic genes in hMSCs [61,77]. Meanwhile, the phosphorylation of ERK 1/2 generates nuclear factor kappa-light-chain-enhancer in activated B cells (NF-κB), which subsequently translocates into the nucleus to increase the production of BMP-2 and integrin β1. BMP-2 binds to the BMP-2 receptor and synthesizes its downstream protein phosphorylated Smad 1/5/8. These proteins translocate into the nucleus and enhance the expression of RUNX2. Both feedback upregulation of integrin β1 and activation of the BMP signalling pathway contribute to the upregulation of RUNX2, resulting in the enhanced expression of osteogenic genes. Secondly, perfusion enhances the endogenous production of vascular endothelial growth factor (VEGF), which binds to the VEGF receptor and subsequently activates the ERK 1/2-RUNX2 signalling pathway in hMSCs [73,88]. Moreover, perfusion upregulates C-FOS, which forms an AP-1 complex with c-Jun to activate the transcription of osteogenic genes [73]. In addition, perfusion activates mechanoreceptor focal adhesion-mediated Rho signalling that upregulates nuclear translocation of the protein tafazzin. This protein interacts with RUNX2 to activate the transcription of osteogenic genes in hMSCs [63]. Furthermore, perfusion activates transient receptor potential vallanoid 4 (TRPV4)-intracellular calcium influx signalling, which produces calcineurin, and in turn promotes translocation of nuclear factor of activated T-cells, cytoplasmic 1 (NFATc1) into the nucleus [64]. NFATc1 and OSX form a complex to induce the transcription of osteogenic genes in hMSCs. 

Vibration may induce osteogenesis in hMSCs via several metabolic routes, such as TRPV 1 channel, BMP signalling pathway, and focal adhesion [28]. Through the TRPV 1 channel, vibration activates protein kinase C and ERK 1/2, resulting in the synthesis of the downstream protein β-catenin. β-catenin translocates into the nucleus and induces the expression of RUNX2, thereby promoting the transcription of osteogenic genes. Meanwhile, vibration also increases the endogenous production of BMP-2, which activates the BMP signalling pathway to upregulate RUNX2, resulting in the enhancement of osteogenesis. Moreover, vibration may activate focal adhesion, which leads to signalling cascades of FAK and ERK 1/2, which are linked to the activation of RUNX2.

Dynamic stretching increases the endogenous production of BMP-2 and VEGF in hMSCs, thus activating RUNX2 via the BMP and VEGF signalling pathways, respectively [48,66]. In addition, dynamic stretching upregulates ligand Jagged1 (JAG1), which subsequently mediates Notch signalling to inhibit the function of histone deacetylase 1 (HDAC1) in blocking the Wnt/β-catenin signalling pathway that is involved in the osteogenesis of hMSCs [65]. Furthermore, dynamic stretching increases the expression of long non-coding RNA H19, which restricts the role of microRNA-138 in blocking the FAK-ERK 1/2-RUNX2 signalling pathway [67]. The osteogenic potential of hMSCs is enhanced when such inhibitory molecules are inactivated by dynamic stretching. 

## 5. Challenges Associated with the Future Translation of hMSC-Derived Bone Grafts to Large Bone Defect Therapy

The existing hMSC-derived bone grafts are not widely used in the clinical treatment of large bone defects due to several limitations. One of the limitations is that the viability of cells at the central core of bone grafts is very low because conventional static culture does not deliver sufficient nutrient and oxygen supplies to the cells situated at the central core of the scaffolds [20,21]. As a result, some clinical trial subjects suffered from a lack of bone healing due to osteolysis and resorption of hMSC-derived bone grafts [18,19]. With the advances in bioreactor technologies, dynamic culture approaches using bioreactors that provide mechanical loading are being developed to solve this concern. For instance, perfusion culture supports the interstitial flow of cell culture medium throughout the porous scaffolds to ensure sufficient nutrient and oxygen is delivered to the cells at the central core of the scaffolds. Several in vivo studies have demonstrated that bioreactor-generated animal-MSC-derived bone grafts induce more new bone formation compared to static-culture-generated bone grafts [21,23,24]. Although mechanically loaded hMSCs have been shown to undergo in vitro and in vivo osteogenesis [8,28], they have not been used for the treatment of bone defects in animal models or clinical studies. Hence, future works should focus on developing more defined strategies of using mechanically loaded hMSC-derived bone grafts in in vivo bone defect studies to evaluate their ability to mediate new bone formation and host tissue integration. Besides the in vitro culture period, it is also essential to keep transplanted hMSC-derived bone grafts viable in a host for a sufficient period in order to mediate host tissue integration and new bone formation. As the host-derived neovascularization occurs slowly in the bone graft, the in vitro vascularization of hMSC-derived bone graft might be required. However, the in vitro vascularization of a large tissue-engineered construct remains challenging to date [89].

Another limitation of the existing hMSC-derived bone grafts is inadequate bone matrix and poor mechanical strength. The application of mechanical loading to the hMSC-laden scaffolds could be important to create a strong and functional bone graft. Extensive studies have shown that physiologically relevant mechanical loadings (e.g., dynamic compression and perfusion) and non-conventional loadings (e.g., vibration and dynamic stretching) are effective in enhancing osteogenesis and bone mineralization in hMSCs [8,26,27,28]. These loadings may make the mechanical properties of the ECM comparable to that of the native bone. However, the mechanical properties of mechanically loaded hMSC-derived bone grafts have not been well-characterized, necessitating more mechanical testing. So far, only one study has demonstrated that mechanical loading improved the Young modulus of hMSC-derived bone grafts from 150 kPa to 270 kPa, but this value is far below from that reported for human bone tissues (1–20 GPa) [45,90]. Therefore, the scaffold’s initial Young modulus should be taken into account in future works by selecting the ideal biomaterials that have similar Young’s modulus to that of native bone tissues.

Moreover, a basic problem for positive clinical translation of the existing hMSC-derived bone grafts is their large-scale production [89]. With the advances in bioreactor technologies, various forms of bioreactors can be used to maintain a well-controlled microenvironment to regulate the expansion of hMSCs and bone tissue development. The advantages of using bioreactors in the development of hMSC-derived bone grafts include: (i) it enhances the functionality of grafts through the application of mechanical loading, (ii) it addresses the diffusional limitations of oxygen and nutrients, (iii) it prevents the accumulation of metabolic waste products, (iv) it can scale-up to clinically relevant size, (v) it provides improved reproducibility and standardization, and (vi) it allows testing the graft’s responses to a range of experimental parameters in a systematic manner [91]. By using bioreactors, the effects of different mechanical loadings on the osteogenesis of hMSCs could be easily compared under similar experimental conditions (e.g., same source of hMSCs, same type of porous scaffold with similar pore size, and same cell culture medium) to determine the most effective loading conditions, especially loading type. 

Numerous studies have shown that some mechanical loadings may share a similar metabolic route in inducing the osteogenesis of hMSCs. For instance, besides the classical pathway (ERK 1/2–RUNX2), perfusion and dynamic stretching can induce osteogenesis via VEGF and BMP signalling pathways [48,61,66,88]. Future studies should develop hybrid bioreactors combining different types of mechanical loading (e.g., perfusion and dynamic stretching or perfusion and vibration) to be applied to hMSCs in order to achieve a synergistic osteogenic effect. Based on mechanotransduction signalling for hMSC osteogenesis, the therapeutic benefits of mechanically loaded hMSCs for bone tissue engineering could be further optimized. Therefore, extensive studies are required in order to fully elucidate the comprehensive mechanism of mechanical loading in osteogenesis.

## 6. Conclusions 

In conclusion, the clinical goal of bone tissue engineering is to repair damaged large bones by avoiding bone injury progression, restoring and maintaining the bone function. This approach combines hMSCs, 3D scaffolds, osteoinductive factors and appropriate mechanical loading to create functional bone grafts. hMSCs have become a desirable source in bone tissue engineering, as they can be easily harvested and undergo osteogenic differentiation. Various types of mechanical loading, such as compression, perfusion, stretching and vibration, were found to enhance the osteogenesis and bone mineralization of hMSCs compared to those of static-cultured hMSCs. Many mechanotransduction signalling pathways for hMSC osteogenesis have been discovered, allowing further optimization of the therapeutic benefits of mechanically loaded hMSCs for bone tissue engineering. Despite the promising outcomes, the mechanical properties of hMSC-derived bone grafts have not been well-characterized, and there is lack of in vivo bone defect studies involving mechanically loaded hMSC-derived bone grafts. Hence, future works should necessitate mechanical testing of mechanically loaded hMSC-derived bone grafts and focus on developing more defined strategies of using mechanically loaded hMSC-derived bone grafts for in vivo bone defect studies. In short, it is envisioned that mechanically loaded hMSCs would be an ideal source for future bone tissue engineering. 

## Figures and Tables

**Table 1:** Summary of the recent studies on effects of mechanical loading towards the osteogenesis of human mesenchymal stem cells (hMSCs) in a 2D planar culture.

Loading	Loading Regime	Substrate	Medium Supplementation	Osteogenesis	Cell Source/Differentiation Status before Mechanical Loading	Ref.
Perfusion	0.276 μL/min (0.12–0.15 dyn/cm^2^) for 4 days; unidirectional flow	Poly-l-lysine-coated glass	Dexamethasone + β-glycerophosphate	↑ RUNX2	Bone marrow/undifferentiated	Kim et al. 2014 [63]
				↑ ALP		
Perfusion	12 dyn/cm^2^ for 3 days; unidirectional flow	Collagen-coated glass	None	↑ OSX	Bone marrow/undifferentiated	Hu et al. 2017 [64]
				↑ ALP		
Perfusion	10 dyn/cm^2^ for 3 weeks; unidirectional flow	Ultrahigh molecular weight polyethylene hybrid nanocomposite-coated glass	Undisclosed	↑ RUNX2	Umbilical cord blood/undifferentiated	Naskar et al. 2018 [47]
				↑ ALP		
				↑ OPN		
				↑ OCN		
				↑ Calcium deposit		
Stretching	0.5 Hz, 4%, 8 h	Type 1 collagen-coated silicone membrane	Dexamethasone + β-glycerophosphate	↑ ALP	Periosteum/osteogenic differentiated (precultured in osteogenic induction medium for 2 weeks)	Lee et al. 2017 [48]
				↑ OCN		
				↑ Calcium deposit		
Stretching	0.5 Hz, 10%, 24 h/day for 14 days	Type 1 collagen-coated silicone membrane	Dexamethasone + β-glycerophosphate	↑ ALP	Mandibular retromolar bone/undifferentiated	Lohberger et al. 2014 [27]
				↑ ON		
				↑ OPN		
				↑ OCN		
				↑ Calcium deposit		
Stretching	0.5 Hz, 10%, 24 h/day for 3 weeks	Type 1 collagen-coated silicon membrane	None	↑ ALP	Bone marrow/undifferentiated	Wang et al. 2017 [65]
				↑ OCN		
Stretching	0.2 Hz, 3%, 4 h/day for 4 days	Fibronectin-coated PDMS membrane	Epigallocatechin-3-gallate	↑ RUNX2	Bone marrow/undifferentiated	Shin et al. 2017 [66]
Stretching	0.5 Hz, 10% 6 h/day for 7 days	Type 1 collagen-coated silicone rubber	None	↑ RUNX2	Bone marrow/undifferentiated	Wu et al. 2018 [67]
				↑ ALP		
				↑ OCN		
				↑ Calcium deposit		
Stretching	5 Hz, 0.9%, 0.5 h/day for 7 days	TiO_2_ nanotubes substrate	None	↑ RUNX2	Bone marrow/undifferentiated	Chang et al. 2019 [68]
				↑ ALP		
				↑ OPN		
				↑ OCN		
				↑ BSP		
Vibration	50 Hz, 0.05–0.9 × *g*, 30 min/day for 5 days	Extracellular matrix (ECM)-coated polystyrene substrate	None	↑ RUNX2	Periodontal ligament/undifferentiated	Zhang et al. 2015 [49]
				↑ OSX		
				↑ ALP		
				↑ OCN		
Vibration	30 Hz, 0.59 × *g*, 45 min/day for 6 weeks	ECM-coated polystyrene substrate	Dexamethasone + β-glycerophosphate	↑ RUNX2	Bone marrow/undifferentiated	Prè et al. 2013 [69]
				↑ ALP		
				↑ ON		
				↑ OPN		
				↑ BSP		
				↑ Calcium deposit		
Vibration	30 – 800 Hz, 0.3 × *g*, 30 min/day for 14 days	ECM-coated polystyrene substrate	Dexamethasone + β-glycerophosphate	↑ RUNX2	Bone marrow/undifferentiated	Chen et al. 2015 [70]
				↑ ALP		
				↑ OPN		
				↑ Calcium deposit		

**Table 2:** Summary of the recent studies on the effects of mechanical loading on hMSCs in a 3D scaffold.

Bioreactor	Loading Regime	Scaffold	Pore Size	Medium Supplementation	Osteogenesis	Cell Source/Differentiation Status before Mechanical Loading	Ref.
Compression	0.22–1.1%, 1 Hz, 4 h/ day for 4 weeks	Polycaprolactone-β tricalcium phosphate (β-TCP)	-	Dexamethasone + β-glycerophosphate	↑ ALP	Bone marrow/undifferentiated	Ravichandran et al. 2017 [26]
					↑ ON		
					↑ OCN		
					↑ Mineral deposit		
Compression	0.4%, 0.1 Hz, 2 h/day for 1 day	Monetite calcium phosphate	200–650 μm	None	↑RUNX2	Bone marrow/undifferentiated	Gharibi et al. 2013 [60]
Compression	5–20%, 1 Hz, 2 h/day for 4 weeks	Polycaprolactone	-	Dexamethasone + β-glycerophosphate	↓ ON	Bone marrow/undifferentiated	Horner et al. 2018 [71]
					↓ Calcium deposit		
Compression	5–10%, 1 Hz, 9 h/day for 6 days	Collagen	64–93 μm	None	↓RUNX2	Bone marrow/undifferentiated	Schreivogel et al. 2019 [72]
					↓ OCN		
Perfusion	3 mL/min (0.2 dyn/cm^2^) for 2 weeks; oscillatory flow	Hyaluronic acid–poly(lactide-co-glycolide) (PLGA)	300 μm	Dexamethasone + β-glycerophosphate	↑ OCN	Bone marrow/undifferentiated	Mitra et al. 2017 [8]
					↑ Calcium deposit		
					↑ BSP (in vivo)		
					↑ Bone volume fraction (ratio of bone volume to total tissue volume)		
					Host tissues were integrated into both the cell-laden and cell-free scaffolds		
Perfusion	1 mL/min (0.161 dyn/cm^2^) for 3 weeks; unidirectional flow	Decellularized porcine bone construct	250–400 μm	Dexamethasone + β-glycerophosphate	↑RUNX2	Bone marrow and fat/undifferentiated	Wu et al. 2015 [44]
					↑ OPN		
					↑ OCN		
					↑ Calcium deposit		
					Bone marrow MSCs > adipose MSCs (↑ RUNX2, ↑ OPN, ↑ ALP and ↑ Calcium deposit)		
Perfusion	Steady flow (0.045 dyn/cm^2^, 2 weeks) + pulsatile flow (0.045–0.134 dyn/cm^2^, 0.5 Hz, 4 h/day for 3 weeks)	Silk fibroin	400–600 μm	Dexamethasone + β-glycerophosphate	↑ OPN	Fat/osteogenic differentiated (precultured in osteogenic induction medium for 3 days)	Correia et al. 2013 [45]
					↑ BSP		
					↑ Young’s modulus of the bone grafts from 150 kPa to 270 kPa		
					↑ Bone volume fraction		
Perfusion	4.2 dyn/cm^2^, 2 h/day for 2 weeks; intermittent unidirectional flow	PLGA	280–450 μm	None	↑RUNX2	Bone marrow/undifferentiated	Liu et al. 2014 [61]
					↑ ALP		
					↑ OCN		
Perfusion	2.5 mL/min (0.0679 dyn/cm^2^) for 2 h; unidirectional flow	Gelatine-coated polyurethane	334 μm	Dexamethasone + β-glycerophosphate	↑RUNX2	Bone marrow/early and late osteogenic differentiated (precultured in osteogenic induction medium for 7 and 15 days, respectively)	Filipowska et al. 2016 [73]
					↑ OPN		
					↑ OCN		
Perfusion	0.3 mL/min (0.0123 dyn/cm^2^) for 3 weeks; oscillatory flow	Cancellous bone powder	200–800 μm	Dexamethasone + β-glycerophosphate	↑RUNX2	Bone marrow/undifferentiated	Le Pape et al. 2018 [74]
					↑ ALP		
Perfusion	100 μm/s (0.493 dyn/cm^2^) for 3 weeks; oscillatory flow	PLGA	100–150 μm	Dexamethasone + β-glycerophosphate	↑ ALP	Bone marrow/undifferentiated	Moser et al. 2018 [75]
					↑ OPN		
					↑ OCN		
Perfusion	1.5 mL/min (0.0182 or 0.0097 dyn/cm^2^) for 3 weeks; unidirectional flow	β-TCP	750 and 1400 μm	Dexamethasone + β-glycerophosphate	750 μm > 1400 μm (↑ ALP and ↑ OPN)	Bone marrow/undifferentiated	Bernhardt et al. 2011 [76]
Perfusion	4.2 dyn/cm^2^ (2 h/day) + 0.34 dyn/cm^2^ (22 h/day) for 2 weeks; intermittent rapid unidirectional flow	PLGA	280–450 μm	Dexamethasone + β-glycerophosphate	↑RUNX2	Bone marrow/undifferentiated	Liu et al. 2011 [77]
					↑ ALP		
					↑ OCN		
Vibration	30 nm amplitude, 1000 Hz for 7 days	Collagen gel	-	None	↑ OSX	Bone marrow/undifferentiated	Tsimbouri et al. 2017 [28]
					↑ ALP		
					↑ OPN		
					↑ OCN		
